# Neuroprotective Effects of a Small Mitochondrially-Targeted Tetrapeptide Elamipretide in Neurodegeneration

**DOI:** 10.3389/fnint.2021.747901

**Published:** 2022-01-17

**Authors:** Nguyen Thanh Nhu, Shu-Yun Xiao, Yijie Liu, V. Bharath Kumar, Zhen-Yang Cui, Shin-Da Lee

**Affiliations:** ^1^Faculty of Medicine, Can Tho University of Medicine and Pharmacy, Can Tho, Vietnam; ^2^Department of Brain and Mental Disease, Shanghai Municipal Hospital of Traditional Chinese Medicine, Shanghai University of Traditional Chinese Medicine, Shanghai, China; ^3^School of Rehabilitation Medicine, Shanghai University of Traditional Chinese Medicine, Shanghai, China; ^4^Institute of Rehabilitation Medicine, Shanghai University of Traditional Chinese Medicine, Shanghai, China; ^5^Engineering Research Center of Traditional Chinese Medicine Intelligent Rehabilitation, Ministry of Education, Shanghai, China; ^6^Department of Medical Laboratory and Biotechnology, Asia University, Taichung, Taiwan; ^7^School of Rehabilitation Medicine, Weifang Medical University, Weifang, China; ^8^Department of Physical Therapy, Graduate Institute of Rehabilitation Science, China Medical University, Taichung, Taiwan; ^9^Department of Physical Therapy, Asia University, Taichung, Taiwan

**Keywords:** Bendavia, brain, mitochondrial, MTP-31, neurodegeneration, SS-31

## Abstract

Neural mitochondrial dysfunction, neural oxidative stress, chronic neuroinflammation, toxic protein accumulation, and neural apoptosis are common causes of neurodegeneration. Elamipretide, a small mitochondrially-targeted tetrapeptide, exhibits therapeutic effects and safety in several mitochondria-related diseases. In neurodegeneration, extensive studies have shown that elamipretide enhanced mitochondrial respiration, activated neural mitochondrial biogenesis *via* mitochondrial biogenesis regulators (PCG-1α and TFAM) and the translocate factors (TOM-20), enhanced mitochondrial fusion (MNF-1, MNF-2, and OPA1), inhibited mitochondrial fission (Fis-1 and Drp-1), as well as increased mitophagy (autophagy of mitochondria). In addition, elamipretide has been shown to attenuate neural oxidative stress (hydrogen peroxide, lipid peroxidation, and ROS), neuroinflammation (TNF, IL-6, COX-2, iNOS, NLRP3, cleaved caspase-1, IL-1β, and IL-18), and toxic protein accumulation (Aβ). Consequently, elamipretide could prevent neural apoptosis (cytochrome c, Bax, caspase 9, and caspase 3) and enhance neural pro-survival (Bcl2, BDNF, and TrkB) in neurodegeneration. These findings suggest that elamipretide may prevent the progressive development of neurodegenerative diseases *via* enhancing mitochondrial respiration, mitochondrial biogenesis, mitochondrial fusion, and neural pro-survival pathway, as well as inhibiting mitochondrial fission, oxidative stress, neuroinflammation, toxic protein accumulation, and neural apoptosis. Elamipretide or mitochondrially-targeted peptide might be a targeted agent to attenuate neurodegenerative progression.

## Introduction

Neurodegeneration is associated with progressive neuron loss, which causes both motor and cognitive symptoms in patients (Brown, 2019). The incidences of neurodegenerative disorders, such as Alzheimer’s, Parkinson’s, and Huntington’s diseases, are increasing alongside the enhanced life expectancy, leading to a considerable number of deaths and disabilities worldwide (Dorsey et al., 2018; Nichols et al., 2019). Therefore, neurodegenerative therapeutics is critical to reducing the social burden of neurodegenerative disorders (Huang et al., 2019). Despite their diverse etiologies, neurodegenerative disorders share common underlying mechanisms, including mitochondrial dysfunction, oxidative stress, neuroinflammation, and toxic protein accumulation, which interact to induce neural dysfunction and cell death (Gireud et al., 2014; Hou et al., 2018; Maldonado et al., 2018). This complicated pathogenesis makes it challenging to find efficient therapies to combat neurodegenerative progression (Fernandez-Ruiz, 2019). In preclinical studies, several compounds have exhibited significant benefits for neurodegenerative disorders but have failed to clearly show treatable abilities in clinical trials (Wang et al., 2018; Gyengesi and Munch, 2020).

Elamipretide (also called Bendavia, SS-31, or MTP-31) has emerged as an efficient targeted therapy for mitochondrial-related diseases consisting of heart failure and kidney disease (Steggall et al., 2017; Bhatt and Butler, 2018; Miyamoto et al., 2020). Several preclinical studies observed that elamipretide could provide therapeutic effects on neurodegeneration (Yang et al., 2009; Wu et al., 2016; Reddy et al., 2018). A study showed that elamipretide could protect dopaminergic neurons against Parkinsonian damage in a mouse model (Yang et al., 2009). Another study reported that elamipretide reduced amyloid-beta (Aβ) accumulation in a cell model of Alzheimer’s disease (Reddy et al., 2018). In addition, elamipretide was suggested to enhance neural mitochondrial functions in a mouse model of cognitive deficits (Wu et al., 2016). These findings suggest that elamipretide may prevent neurodegenerative disorders. However, evidence from each study makes it difficult to understand the neuroprotective effects of elamipretide comprehensively. In this regard, our review was carried out to systematically summarize the benefits of elamipretide therapy on the underlying mechanisms of neurodegeneration.

We searched the PubMed, Web of Science, and EMBASE databases through May 2021 to find relevant articles using a combination of terms: (“elamipretide” OR “SS-31” OR “MTP-131” OR “Bendavia”) AND (“brain” OR “neur*” OR “cerebral”). Original studies that mentioned the effects of elamipretide on neurodegenerative disorders were included. In addition to particular disease models (e.g., Alzheimer’s, Parkinson’s, and Huntington’s diseases and aging), we included neurodegenerative mechanism-related models, such as neuroinflammation, mitochondrial dysfunction, oxidative stress, and neural apoptosis models. Non-English publications, conference abstracts, reviews, and protocol papers were excluded. Fourteen studies that met the eligibility criteria were included in this review. The characteristics of those studies are summarized in Table 1.

**TABLE 1:** Characteristics of the reviewed studies.

Study	Model	Number	Intervention	Outcomes
Wu et al. (2015b)	Parkinson’s disease (*in vivo*: MPTP 3-month-old C57 mice model; *in vitro:* SN4741 dopamine cells)	*In vivo:* 10 mice/group, four groups (for TH-positive staining): 1. Control 2. MPTP 3. MPTP + elamipretide 4. MPTP + SS-20	*In vivo*: elamipretide 1 mg/kg, i.p, before each MPTP injection 30 min, 1 and 12 h after the final MPTP injection. *In vitro*: Elamipretide 1 and 10 nM.	*In vivo*: protect the TH-positive dopaminergic neurons. *In vitro*: ↓ apoptotic cells (%).
Guo et al. (2013)	Senescence model (8-month-old SAMP8 mice)	6 mice/group, three group 1. SAMR1 (normal mice) + saline 2. SAMP8 + saline 3. SAMP8 + elamipretide	Elamipretide 5 mg/kg, i.p,	Oxidative stress: ↓ ROS, ↑ mRNA and nuclear protein levels of Nrf2 and HO-1 Neuroinflammation: ↓ IL-1β mRNA.
Zhao et al. (2005)	Cognitive impairment (7-day-old SD rats with Isoflurane anesthesia)	24 rats/group, four groups 1. control + PBS 2. control + elamipretide 3. isoflurane + PBS 4. isoflurane + elamipretide	Elamipretide 5 mg/kg, i.p, before inhaling isoflurane	Mitochondria: normalized the ultrastructural abnormalities, density and length. Oxidative stress: ↓ ROS and MDA, ↑ SOD and SOD2. Neural apoptosis: ↓TUNEL-positive cells and caspase-3 positive cells.
Wu et al. (2016)	Cognitive impairment (15-month-old male C57BL/6 mice with Isoflurane anesthesia)	14 mice/group, four groups 1. control + PBS 2. control + elamipretide 3. isoflurane + PBS 4. isoflurane + elamipretide	Elamipretide 5 mg/kg, i.p, before inhaling isoflurane	Mitochondria: ↑ complex I activity, ATP, and MMP levels, ↓ mPTP opening. Oxidative stress: ↓ ROS. Protective pathway: ↑BDNF and p-TrkB; ↑ NMDA-CaMKII-CREB signaling (increased NR2B, CaMKIIα, and CaMKIIβ levels).
Yin et al. (2016)	Cognitive impairment (15-month-old male C57BL/6 mice with Isoflurane anesthesia)	18 mice/group, four groups 1. control + PBS 2. control + elamipretide 3. isoflurane + PBS 4. isoflurane + elamipretide	Elamipretide 5 mg/kg, i.p, before inhaling isoflurane	Mitochondria: ↑ ATP production. Oxidative stress: ↓ ROS levels. Neuroinflammation: ↓NLRP3, cleaved caspase 1, IL-1β, and TNF-α. ↑ IκBα. Neural apoptosis: ↓ cytochrome c, activated-caspase-3, and Bax; ↑ Bcl-2.
Guedes-Dias et al. (2016)	Neuroinflammatory model (10–11-week-old male C57BL/6 mice with lipopolysaccharide injection.)	24 mice/group, four groups 1. control + placebo 2. control + elamipretide 3. LPS + placebo 4. LPS + elamipretide	Elamipretide 5 mg/kg, i.p, before 30 min and daily 3 days after LPS injection.	Mitochondria: ↑MMP and ATP. Oxidative stress: ↓ ROS and MDA. Neuroinflammatory: ↓TNF-α and IL-6. Neural apoptosis: ↓ TUNEL-positive cell in DG and CA1. Neuroprotective pathway:↑ BDNF protein and p-TrkB/TrkB ratio.
Chen et al. (2012)	Neuroinflammatory model (20-month-old Wistar male rats with lipopolysaccharide injection.)	240 20-month-old Wistar male rats, four groups: 1. vehicle (*n* = 50) 2. Elamipretide (*n* = 50) 3. LPS injection (*n* = 70) 4. LPS injection + Elamipretide (*n* = 70)	Elamipretide 5 mg/kg, i.p, 30 min before LPS injection.	Neuroinflammatory: ↓TNF-α and IL-1β and reduce astrocyte activation.
Harland et al. (2020)	Pyroptosis mice model (15-month-old C57BL/6 male mice with isoflurane anesthesia)	6 mice/group, four groups: 1. control + normal saline 2. control + elamipretide 3. isoflurane + normal salin 4. isoflurane + elamipretide	Elamipretide 5 mg/kg, i.p, 30 min before isoflurane anesthesia and then one time per day for three consecutive days	Mitochondria: ↑MMP and ATP, ↓ abnormal mitochondria, Drp1 activities Neuroinflammation: ↓NLRP3, cleaved caspase 1, IL-1β, and IL-18. Neural apoptosis: ↓ TUNEL positive-cells
Díaz-Hung and González Fraguela (2014)	Neuroinflammatory model (lipopolysaccharide -treated microglial BV-2 cells)	*In vitro* study	Elamipretide 100 nM	Oxidative stress: ↓ ROS levels. Neuroinflammation: ↓ COX-2 and iNOS. Mitochondria: ↑ mitochondrial length and Fis-1 protein levels.
Reddy et al. (2018)	Alzheimer’s disease (Mutant AβPP on N2a cells cells)	*In vitro* study	Elamipretide 0.25 nM	Mitochondria: ↑ mDNA number, TOM-20, ATP, and cytochrome c oxidase activity; ↓ GTPase Drp1 activities. Oxidative stress: ↓ hydrogen peroxide production and lipid peroxidation. Neural apoptosis: ↓ apoptotic cells. Protein accumulation: ↓ Aβ40 and Aβ42.
Calkins et al. (2011)	Alzheimer’s disease (Mutant AβPP on N2a cells cells)	*In vitro* study	Elamipretide 1 nM	Mitochondria: ↑ mRNA expression of complex I (NADH-3), complex IV (COX-3); ↑ATP. ↑ mRNA expressions of Mfn2 and Opa1; ↓ mRNA expressions of Drp-1 and Fis-1; ↓ mitochondrial fragmentation and swelling. Oxidative stress: ↑ antioxidant enzyme Peroxiredoxins (1-6) and H_2_O_2_ production.
Xie et al. (2013)	Alzheimer’s disease (Mutant AβPP on N2a cells cells)	*In vitro* study	Elamipretide (not given the concentration)	Mitochondria: ↑ mitochondrial motility, frequency and the length.
Sweeney and Song (2016)	Huntington’s disease (mutant Htt, STHdh Q111/Q111)	*In vitro* study	Elamipretide 2.5 nM	Mitochondria: ↓ mRNA and protein expressions of Drp-1, Fis-1; ↑ mRNA and protein expressions of MFN-1, MFN-2 and OPA-1. ↑mRNA and protein expressions of PGC1α, PGC1β, Nrf1, Nrf2 and TFAM. ↑ complex I, IV, and V, ATP, ↓ GTPase Drp1 activity. Increased the mitochondrial number. Oxidative stress: ↓ hydroperoxide, lipid peroxidation ↑ cell viability
Zuo et al. (2020)	Neural oxidative stress neurons model (tert-butyl hydroperoxide in SH-SY5Y and N2A cell lines)	*In vitro* study	Elamipretide 1 nM	Mitochondria: ↓ mitochondrial depolarization, ↑ NADH- dehydrogenase activity. Oxidative stress: ↓ lipid peroxidation and ROS Neural apoptosis: ↓ apoptotic cells, caspase-9 levels

*SOD, Superoxide dismutase; Nrf2, Nuclear factor erythroid 2-related factor 2; HO-1, Heme oxygenase 1; ROS, reactive oxidative species; MDA, Malondialdehyde; MMP, mitochondrial membrane potential; mPTP, Mitochondrial permeability transition pore; BDNF, Brain-derived neurotrophic factor; TrkB, Tropomyosin receptor kinase B; NMDA, N-methyl-D-aspartate receptor; CaMKII, Ca^2+^/calmodulin-dependent protein kinase II; CREB, cAMP response element binding protein; NLRP3, NLR family pyrin domain containing 3; TOM, translocate of the outer membrane; NADH, Nicotinamide adenine dinucleotide; COX, Cytochrome c oxidase; PGC-1α, peroxisome proliferator-activated receptor gamma coactivator 1-alpha; NRF-1/2, nuclear respiratory factor 1 and 2; TFAM, mitochondrial transcription factor A; OPA1, Dynamin-like 120 kDa protein; MFN, Mitofusin; Dpr1, Dynamin-related protein-1; Fis-1, Mitochondrial fission 1 protein; MPTP, 1-methyl-4-phenyl-1,2,3,6-tetrahydropyridine; SAMR1, senescence accelerated resistant 1 mice; SAMP8, senescence-accelerated mouse prone 8.*

## Characteristics of Elamipretide

**FIGURE 1:**
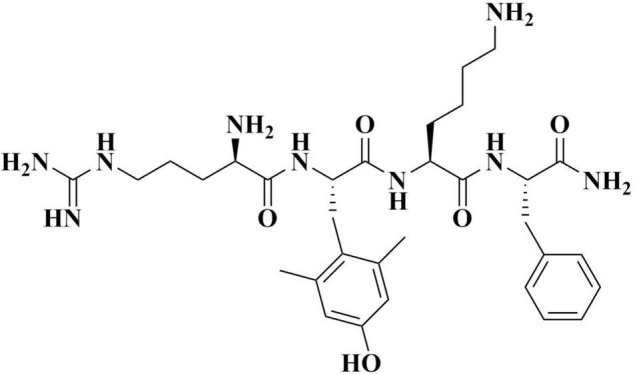
The molecular structure of elamipretide.

Elamipretide (C_32_H_49_N_9_O_5_; Figure 1) is a water-soluble tetrapeptide with a small molecular weight (Szeto and Birk, 2014). Evidence has shown that elamipretide can cross the brain-blood barrier (Michael Seganish et al., 2017), exhibiting high permeability in neural cells (Szeto, 2014; Szeto and Birk, 2014). Elamipretide is rapidly absorbed by mitochondria which is independent of the mitochondrial membrane potential (Dai et al., 2017). Once entering mitochondria, elamipretide mainly concentrates at the inner mitochondrial membrane, where the electron transport system is located (Zhao et al., 2004; Chavez et al., 2020).

Recent evidence suggests that the benefits of elamipretide on mitochondria-related diseases are primarily based on its binding with cardiolipin in the inner mitochondrial membrane (Szeto and Birk, 2014). Of note, cardiolipin is important for crista formation, contributing to the optimal activities of the electron transport system for producing ATP (Szeto, 2014). In addition, cardiolipin supports cytochrome *c* in conveying electrons from complex III to complex IV in mitochondrial respiration stage 3 (Szeto, 2014; Yeagle, 2016). When cardiolipin is excessively oxidized, cytochrome *c* is detached from the inner mitochondrial membrane, inducing electron transport impairment and mitochondria-mediated neural apoptosis (Szeto and Birk, 2014). Elamipretide binds with cardiolipin to facilitate the interaction of cytochrome *c* and cardiolipin, thereby stabilizing the mitochondrial respiratory system against the severe effects of various disease models (Dai et al., 2017; Michael Seganish et al., 2017).

## The Neuroprotective Effects of Elamipretide on Neurodegeneration

### Elamipretide and Mitochondrial Dysfunction

In neurodegenerative disorders, neural mitochondrial dysfunction is the hallmark mechanism induced by both genetic causes (primary mitochondrial dysfunction) and non-genetic causes (secondary mitochondrial dysfunction) (Jellinger, 2010). Neural mitochondrial dysfunction is characterized by impaired mitochondrial respiration that includes specific inhibition of the electron transport system and ATP depletion (Johri and Beal, 2012). In addition, neural mitochondrial quality-control, including mitochondrial biogenesis, dynamics, and mitophagy (autophagy of mitochondria), is dysregulated in neurodegeneration (Jellinger, 2010; Yan et al., 2020). Neural mitochondrial biogenesis is inactivated, as evidenced by reductions in biogenesis regulators (e.g., PGC-1α and SIRT-1) and mitochondrial membrane translocators (e.g., TOM and TIM), leading to a reduction in the number of healthy mitochondria (Yan et al., 2020). The neural mitochondrial dynamics in neurodegenerative disorders are imbalanced and characterized by reductions in mitochondrial fusion and increases in mitochondrial fission (Yan et al., 2020). Downregulation of neural mitophagy is also observed in neurodegenerative disorders, augmenting dysfunctional mitochondrial accumulation (Yan et al., 2020). Neural mitochondrial respiratory impairment and quality-control dysregulation promote progressive neuron loss (Pathak et al., 2013). Hence, mitochondrial function is hypothesized to be a promising therapeutic target for neurodegeneration. As a mitochondria-targeted compound, elamipretide has been shown to protect neural mitochondrial functions against mechanisms involved in neurodegeneration, including toxic protein aggregation, neuroinflammation, and neural oxidative stress.

#### Elamipretide Protects Neural Mitochondrial Functions Against Toxic Protein Aggregation in Neurodegeneration

In Alzheimer’s disease, previous studies showed that Aβ protein progressively accumulates in neural mitochondria, impairing the mitochondrial electron transport system, especially complex I, complex III, and complex IV (Calkins et al., 2011; Sorrentino et al., 2017). In addition, the neural mitochondrial membrane potential has been showed to be reduced by Aβ toxicity, disturbing the electron flux passing through mitochondrial complexes in mouse model of Alzheimer’s disease (Xie et al., 2013). Aβ protein accumulation consequently results in ATP reduction in neurons (Letellier et al., 1994).

One study used N2a cells incubated with the Aβ (25–35) peptide (30 μM), which mimicked the neurons with toxic Aβ protein accumulation in Alzheimer’s disease (Manczak et al., 2010). This study showed that the mRNA expressions of complex I (NADH-sub3) and complex IV (COX3) were reduced in the Aβ-treated N2a cells compared to those in normal N2a cells (Manczak et al., 2010). By contrast, treatment with elamipretide (1 nM) significantly increased those mRNA levels (NADH-sub3: 6.6-fold; COX3: 7.3-fold) in the Aβ-treated N2a cells, suggesting that elamipretide might normalize the neural mitochondrial electron transport chain against Aβ toxicity in Alzheimer’s disease (Manczak et al., 2010). The cytochrome oxidase activity, mitochondrial membrane potential, and ATP production in the Aβ-treated N2a cells were also enhanced after elamipretide treatment (Manczak et al., 2010). Regarding mitochondrial quality-control, in an *in vitro* study that used the mutant AβPP cDNA clone transfected into N2a cells, the mitochondrial DNA copy number and TOM-20 protein level were reduced in the mutant AβPP-translated N2a cells compared to normal N2a cells, whereas those levels were enhanced by elamipretide treatment (20 μM), upregulating neural mitochondrial biogenesis against Aβ aggregation in neural cells (Reddy et al., 2018). Despite different models and dosages of elamipretide, the two mentioned studies have reported that elamipretide enhanced mitochondrial fusion as well as reduced mitochondrial fission in neurons against Aβ toxicity, as evidenced by the increased levels of mitochondrial fusion factors (i.e., Mfn1, Mfn2, and Opa1) and the reduced levels of Drp1 and Fis-1 (Manczak et al., 2010; Reddy et al., 2018). Furthermore, another study used neurons from the AβPP Tg2576 mouse model, showing that the length and number of neural mitochondria per neurite were reduced compared to normal neurons, whereas elamipretide treatment could reduce those levels in the AβPP cells (Calkins et al., 2011). This finding might indirectly imply that elamipretide might activate neural mitophagy to reduce dysfunctional mitochondria in neurons with Aβ toxicity (Calkins et al., 2011). Collectively, elamipretide treatment appears to recover neural mitochondrial respiration and neural mitochondrial quality-control in cell models of Aβ accumulation, attenuating the damage of Aβ toxicity on neural mitochondria. However, each study mentioned above just investigated the specific aspects of mitochondrial functions, making the findings summarized hereby inconclusive and requiring additional research. Besides, there is a lack of evidence in the effects of elamipretide on neural mitochondrial biogenesis regulators (e.g., PCG1-α, TFAM, NRF1, and NRF2) under Aβ toxicity. It should be noted that the activation of biogenesis regulators, especially PCG1-α, have been proven to attenuate not only neural mitochondrial dysfunction but also cognitive impairment in Alzheimer’s disease (Sweeney and Song, 2016). Further studies need to investigate the effects of elamipretide on biogenesis regulators to clearly comprehend the therapeutic mechanisms of elamipretide.

In Huntington’s disease, mutant Huntington (mHtt) protein aggregation induces neural mitochondrial deficits, characterized by the specific inactivation of mitochondrial complexes II, III, and IV (Carmo et al., 2018). Evidence has shown that mHtt protein not only impairs mitochondrial biogenesis regulators but also interacts with mitochondrial translocation factors, dysregulating mitochondrial quality control (Guedes-Dias et al., 2016). In a previous study using mHtt striatal progenitor neurons of Huntington’s disease, elamipretide treatment (2.5 nM) enhanced the mRNA levels of complex I (ND1; 1.4-fold), complex IV (COX-1, COX-2, and COX-3; 1. 4-, 1. 6-, and 1.5-fold, respectively), and complex V (ATP-6; 1.4-fold), increasing ATP production against mHtt protein accumulation (Yin et al., 2016). This study also provided that elamipretide treatment increased the mRNA levels of biogenesis regulators (i.e., PGC-1α, PGC-3β, and TFAM) and the number of neural mitochondria in those cells, thereby attenuating mHtt-suppressed neural mitochondrial biogenesis (Yin et al., 2016). Moreover, the levels of mitochondrial fusion factors (Mfn1, Mfn2, and Opa1) were upregulated, and the levels of mitochondrial fission factors (Fis1 and Drp1) were downregulated by elamipretide treatment, implying that neural mitochondrial dynamics were rebalanced by elamipretide (Yin et al., 2016). These results proved that elamipretide attenuates neural mitochondrial dysfunction induced by mHtt protein accumulation in Huntington’s disease. However, in this study (Yin et al., 2016), elamipretide treatment did not change the expressions of some other electron transport chain genes (i.e., ND3, ND6, and Cyt B), suggesting that elamipretide might affect specific targets in the electron transport system to protect neural mitochondrial respiratory functions against Htt toxicity. This finding raised the need for clarifying targeted and off-targeted effects of elamipretide in neurodegeneration. Besides, the effects of elamipretide on neural mitophagy in HD have also maintained unclear, which might be a concern for further studies.

#### Elamipretide Attenuates Neuroinflammation-Induced Neural Mitochondrial Dysfunction

Neuroinflammation is both a cause and a consequence of neural mitochondrial dysfunction in neurodegeneration (Jellinger, 2010; Harland et al., 2020). A previous study conducted elamipretide treatment (5 mg/kg, i.p.) in 10–11-week-old mice with neuroinflammation induced by a 2 μL lipopolysaccharide injection into the lateral ventricle (Zhao et al., 2019). This study showed that the level of ATP production and the mitochondrial membrane potential in the hippocampus of neuroinflammatory mice were reduced compared to those in normal mice, whereas those levels in neuroinflammatory mice were significantly enhanced by elamipretide treatment (Zhao et al., 2019). Other studies analyzed the effects of elamipretide on neuroinflammation induced by isoflurane administration in aged rodent models (Wu et al., 2015a,^2016^). In those studies, isoflurane administration reduced neural mitochondrial complex I activities, mitochondrial membrane potential, and ATP production in the hippocampus, whereas elamipretide pretreatment (5 mg/kg, i.p.) recovered those levels to normal (Wu et al., 2015a,^2016^; Zuo et al., 2020). Elamipretide treatment (5 mg/kg, i.p.) has been proven to suppress the activities of Drp1 (mitochondrial fission factor) and reduce the number of abnormal hippocampal mitochondria in neuroinflammatory mice (Zuo et al., 2020).

In summary, using the different neuroinflammatory models with the same dosage of elamipretide (5 mg/kg, i.p.) *in vivo*, the available studies suggested that elamipretide might be able to attenuate neural mitochondrial dysfunction induced by neuroinflammation in neurodegeneration. Of note, although the lipopolysaccharide injection model is useful to mimic neuroinflammation in many neurodegenerative disorders such as Alzheimer’s disease, Parkinson’s disease, and Huntington’s disease, the variety of administrative protocols of this preclinical model might induce “a plethora of results” (Batista et al., 2019), and thereby might make the effects of elamipretide observed here misleading. Therefore, we need to make a caution for the finding summarized here.

#### Elamipretide Attenuates Oxidative Stress-Induced Neural Mitochondrial Dysfunction

Neural mitochondrial dysfunction is noted as the main causative factor of neural oxidative stress, and oxidative stress conversely deteriorates mitochondrial dysfunction in neurodegenerative progression (Guo et al., 2013; Stepien et al., 2017). A study investigated the effects of elamipretide on a neuron cell line (N2a cells) cultured with tert-butyl hydroperoxide, which is commonly used as an *in vitro* model to mimic neural oxidative stress (Zhao et al., 2005). In this study, NADPH dehydrogenase activity and the mitochondrial membrane potential were reduced in tert-butyl hydroperoxide-treated N2a cells. In contrast, those levels were increased after elamipretide treatment (1 nM), suggesting that elamipretide treatment might protect neural mitochondrial functions against neural oxidative stress (Zhao et al., 2005).

**FIGURE 2:**
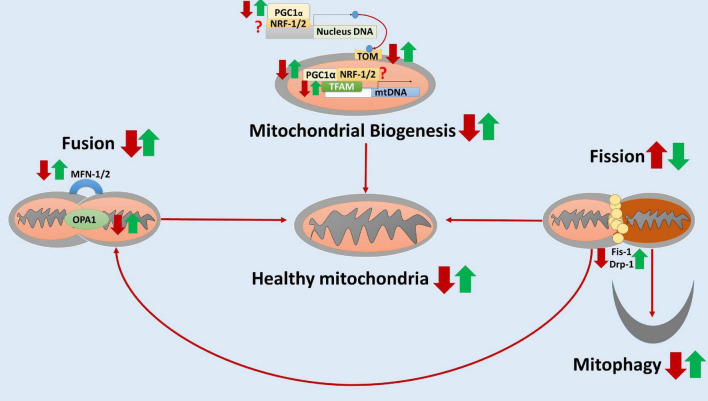
The hypothesized diagram summarizes the neuroprotective effects of elamipretide on neural mitochondria quality-control. In neurodegeneration, neural mitochondrial biogenesis regulators (e.g., PCG-1α, TFAM) and the translocate factors (e.g., TOM) are reduced, suggesting the inactivation of neural mitochondrial biogenesis. Besides, the mitochondrial fusion is inhibited and mitochondrial fission is overactive, as evidenced by the reduced levels of MNF-1, MNF-2, and OPA1 as well as the increased levels of Fis-1 and Drp-1. Moreover, mitophagy is reduced, augmenting the accumulation of dysfunctional mitochondria in neurodegenerative disorders. Elamipretide appears to enhance neural mitochondrial biogenesis regulators and fusion, inactivate neural mitochondrial fission. Moreover, elamipretide has been shown to promote neural mitophagy, increasing the healthy mitochondria number and thus enhancing mitochondrial respiration in the nervous system. the red arrows, the effects of neurodegeneration; the green arrows, the effects of elamipretide on neurodegeneration; ?, unknown.

Taken together, these studies showed that elamipretide might protect neural mitochondrial respiration and neural mitochondrial quality-control against the further damages of toxic protein accumulation, neuroinflammation, and neural oxidative stress. Hence, we hypothesized that elamipretide could prevent secondary neural mitochondrial dysfunction in neurodegeneration. A hypothesized diagram was drawn to summarize the integrated findings (Figure 2).

### Elamipretide and Neural Oxidative Stress

Oxidative stress contributes to neurodegenerative disorders characterized by oxidant overproduction and antioxidant inhibition (Stepien et al., 2017). Evidence has shown that oxidative stress occurs in the early stage of Alzheimer’s and Parkinson’s diseases, implying that oxidative stress might be a fundamental mechanism in neurodegeneration (Díaz-Hung and González Fraguela, 2014). In addition, neural oxidative stress is augmented by other neurodegenerative mechanisms, such as protein accumulation and neuroinflammation (Jellinger, 2010). Thus, it is critical to examine whether elamipretide treatment prevents both primary and secondary oxidative stress in neurodegeneration.

#### Elamipretide Prevents Neural Primary Oxidative Stress

In one previous study, the anti-oxidant effects of elamipretide were evaluated by using N2a and SH-SY5Y cells cultured with 25–50 μM tert-butyl hydroperoxide. Compared to control cells, the levels of cytotoxicity (measured in lactate dehydrogenase release), 4- lipid peroxidation (measured in Hydroxy-2-nonenol), and intracellular ROS production in cultured cells increased. In contrast, treatment with elamipretide (0.1 and 1 nM) reduced those levels, implying that elamipretide directly prevents primary oxidative stress in neurons (Zhao et al., 2005). This study also showed that the effects of elamipretide on neural oxidative stress might depend on the dosage, which needs to be noted for further preclinical and clinical studies.

#### Elamipretide Prevents Secondary Oxidative Stress Induced by Other Mechanisms in Neurodegeneration

In Alzheimer’s disease, evidence indicates that accumulated Aβ protein inserts into the cellular bilayer and induces oxidative stress, which suppresses the level of antioxidant peroxiedoxin and increases the levels of ROS, hydrogen peroxide, and lipid peroxidation (Guo et al., 2013). One study showed that, in the N2a cells with Aβ toxicity mimicking neurons in Alzheimer’s disease, peroxiredoxin (Prx 1 to 6) mRNA expression was reduced in comparison with normal N2a cells, whereas treatment with elamipretide (1 nM) was shown to increase this level in the Aβ-treated N2a cells (Manczak et al., 2010). In addition, the levels of hydrogen peroxide and lipid peroxidation were significantly reduced by elamipretide treatment (1 nM) (Manczak et al., 2010; Reddy et al., 2018). Regarding Huntington’s disease, the levels of hydrogen peroxide and lipid peroxidation were increased in mutant Htt neurons compared to normal neurons, whereas the levels of hydrogen peroxide and lipid peroxidation were significantly reduced after elamipretide treatment (2.5 nM) (Yin et al., 2016). In summary, elamipretide might reduce toxic protein accumulation-induced neural oxidative stress in neurodegenerative disorders, as observed in Aβ and mHtt toxicity models.

Neuroinflammation is a common feature leading to increased oxidative stress by activating the release of ROS in neurodegeneration (Fischer and Maier, 2015). Previous studies have suggested that elamipretide may counteract neuroinflammation to reduce neural oxidative stress (Wu et al., 2015a,^2016^, ^2017^; Mo et al., 2019; Zhao et al., 2019). In one study that used a mouse model with a 2 μL lipopolysaccharide injection, elamipretide treatment (5 mg/kg, i.p.) normalized the ROS levels in the brain (Zhao et al., 2019). In a mouse model of isoflurane administration-induced neuroinflammation, studies reported that elamipretide treatment reduced the isoflurane-augmented ROS level and increased isoflurane-suppressed superoxide dismutase (SOD) activity in the inflammatory hippocampus (Wu et al., 2015a,^2016^, ^2017^). Additionally, elamipretide treatment was proven to reduce the oxidative response of inflammation-activated microglia, which is considered the main source of neural oxidative stress (Mo et al., 2019).

Another study investigated the effects of elamipretide in the Senescence accelerated mouse prone 8 (SAMP8) aging model in which Aβ protein accumulation and oxidative stress are prominent pathologies (Hao et al., 2017). The Nrf2-ARE pathway, which plays a key role in neural oxidative stress, has been observed to be activated in the hippocampus of SAMP8 mice by elamipretide treatment (5 mg/kg i.p.) in comparison with SAMP8 mice without treatment, as evidenced by increases in the gene expressions of Nrf2 and HO-1 (Hao et al., 2017). Elamipretide also suppressed the level of ROS in the hippocampal tissue of SAMP8 in this study (Hao et al., 2017). Of note, aging might promote neural oxidative stress in the brain, developing the aging-related neurodegenerative disorders (e.g., Alzheimer’s disease and Parkinson’s disease) (Chen et al., 2012). Elamipretide could prevent neural oxidative stress against aging, thereby might reduce the risks of neurodegeneration in aging.

In brief, both *in vivo* and *in vitro* studies suggest that elamipretide may reduce not only the primary oxidative stress but also the secondary oxidative stress augmented by toxic protein accumulation, neuroinflammation, and aging. Thus, we hypothesized that elamipretide might prevent oxidative stress and is involved in other mechanisms of neurodegenerative disorders.

### Elamipretide and Neuroinflammation

The neuroinflammatory process is a common mechanism in neurodegenerative disorders (Gilhus and Deuschl, 2019). In the brain, neuroinflammatory stimuli activate microglia and astrocytes to release inflammatory cytokines and induce neuroinflammation (Jellinger, 2010). Remarkably, the transcription and release of cytokines in neuroinflammation are controlled by two major pathways: the endoplasmic reticulum/Golgi pathway (e.g., TNF and IL-6) and the caspase-mediated inflammatory pathway (e.g., NLRP3, caspase-1, and IL-1β) (Groslambert and Py, 2018). Evidence has indicated that neuroinflammation is strongly associated with synaptic dysfunction, neurogenesis inhibition, and neural cell death (Lymana et al., 2014).

There are a limited number of studies supporting the anti-inflammatory effects of elamipretide in neurodegenerative disorders. In a neuroinflammatory model induced by lipopolysaccharide injection (2 μL) into the lateral ventricle, elamipretide treatment (5 mg/kg i.p.) reduced the TNF-α and IL-6 protein levels in the lipopolysaccharide-damaged hippocampus (Zhao et al., 2019). Likewise, one study using the same animal model showed that lipopolysaccharide exposure increased the protein levels of hippocampal IL-1β and TNF-α, whereas elamipretide (5 mg/kg i.p) reduced those levels in the hippocampus of lipopolysaccharide-injected aged rats (Liu et al., 2021).

In aged mouse models (15-month-old) with isoflurane anesthesia, two studies showed that the protein levels of NLRP3, caspase-1, and IL-1β were increased after isoflurane exposure, whereas treatment with elamipretide (5 mg/kg, i.p.) reduced those levels in the hippocampal cells (Wu et al., 2015a; Zuo et al., 2020). Similarly, one study using the Sepsis-associated encephalopathy mouse model showed that elamipretide (5 mg/kg, i.p.) also reduced the levels of NLRP3 and IL-1β in the hippocampus of the encephalopathy mice (Wu et al., 2015b). By using different models of neuroinflammation with the same dosage of elamipretide (5 mg/kg, i.p.), those studies suggested that elamipretide could suppress the caspase-mediated inflammatory pathway to reduce neuroinflammation in neurodegeneration.

In aging, toxic proteins might accumulate to develop several neurodegenerative diseases and activate the neuroimmune system to increase neuroinflammation (Schain and Kreisl, 2017). One study using the SAMP8 mouse model of aging showed that elamipretide treatment (5 mg/kg/day, i.p.) could reduce the IL-1β protein level in the brains of SAMP8 mice with Aβ accumulation (Singh et al., 2016), suggesting that elamipretide might protect the brain against the damage of aging.

Regarding the *in vitro* model, elamipretide treatment was also proven to mediate microglia, as evidenced by reductions in the release of the proinflammatory cytokines COX-2 and iNOS from the lipopolysaccharide-activated BV-2 cell model, suggesting that elamipretide treatment could reduce microglial activation in neuroinflammation (Mo et al., 2019).

### Elamipretide and Toxic Protein Aggregation

Aβ, mHtt, or another toxic protein aggregation is the most specific pathology in several neurodegenerative diseases, such as Alzheimer’s and Huntington’s diseases (Jellinger, 2010). Several lines of evidence have confirmed that accumulation of toxic proteins further activates neural mitochondrial dysfunction, neural oxidative stress, and neuroinflammation in neurodegeneration (Jellinger, 2010; Currais et al., 2017). However, previous findings have shown that the toxic protein accumulation also results from neuroinflammatory cascades or neural mitochondrial dysfunction (Jellinger, 2010; Alasmari et al., 2018).

To the best of our knowledge, only one study has evaluated the effect of elamipretide on protein accumulation in neurodegeneration. In this study, significant reductions in Aβ40 and Aβ42 accumulation in an AβPP-translated N2a cell model of Alzheimer’s disease were observed after treatment with elamipretide (0.25 nM) (Reddy et al., 2018). Further preclinical studies need to be conducted to investigate the effects of elamipretide on protein accumulation in several models of neurodegenerative disorders.

### Elamipretide and Neural Apoptotic/Survival Signaling

Neural apoptosis is controlled by neural apoptotic pathways, including caspase-dependent and caspase-independent apoptotic pathways (Cregan et al., 2004). In neurodegenerative disorders, apoptotic pathways are promoted by mitochondrial dysfunction, oxidative stress, neuroinflammation, and protein accumulation (Cregan et al., 2004). In addition, evidence has indicated that neural survival pathways, including the BDNF/TrkB pathway, are suppressed in neurodegeneration (Jellinger, 2010).

In the *in vitro* models of Alzheimer’s disease, elamipretide treatment (1 nM) was found to reduce apoptotic neural cells and enhance neural cell survival in both Aβ-incubated cell and mutant Aβ-transfected cell models (Manczak et al., 2010; Reddy et al., 2018). In another study that used 1-Methyl-4-Phenyl-1,2,3,6-Tetrahydropyridine (MPTP)-induced neurotoxicity mouse model of Parkinson’s disease, elamipretide treatment dose-dependently reduced the percentage of apoptotic dopaminergic cells in the Parkinsonian mice *in vivo* as well as SN4741 dopamine cells treated with MPP^+^ (100 μM) *in vitro* (Yang et al., 2009). In addition, another study showed that neural cell survival in mHtt striatal progenitor neurons of Huntington’s disease was significantly increased after treatment with elamipretide (2.5 nM) (Yin et al., 2016).

The antiapoptotic effects of elamipretide were also reported in an oxidative stress model, as evidenced by reductions of the percentage of neural apoptotic cells and caspase-9 levels in tert-butyl hydroperoxide-treated SH-SY5Y and N2a cell lines (Zhao et al., 2005). In inflammation-activated neural apoptotic models, a previous study showed that elamipretide treatment (5 mg/kg, i.p.) reduced TUNEL-positive neural apoptotic cells (Zhao et al., 2005, 2019; Zuo et al., 2020). Likewise, TUNEL-positive cells, Bax, cytochrome c, and caspase-3 levels were reduced, and the Bcl-2 level was increased by elamipretide treatment in an isoflurane administration model (Wu et al., 2015a,^2017^).

Regarding the neural survival pathway, elamipretide was observed to activate the BDNF pathway against neuroinflammation, as evidenced by increases in the BDNF and p-TrkB/TrkB protein levels in animal models of lipopolysaccharide (Zhao et al., 2019) or with isoflurane administration (Wu et al., 2016). Of note, the BDNF/TrkB survival pathway is considered the main pathway that activates the PI3K/AKT pathway and can inhibit apoptotic pathways to reduce neural apoptosis and enhance neural survival (Jin, 2020). Elamipretide can activate the BDNF/TrkB pathway and thus improve neural survival in neurodegenerative disorders.

**FIGURE 3:**
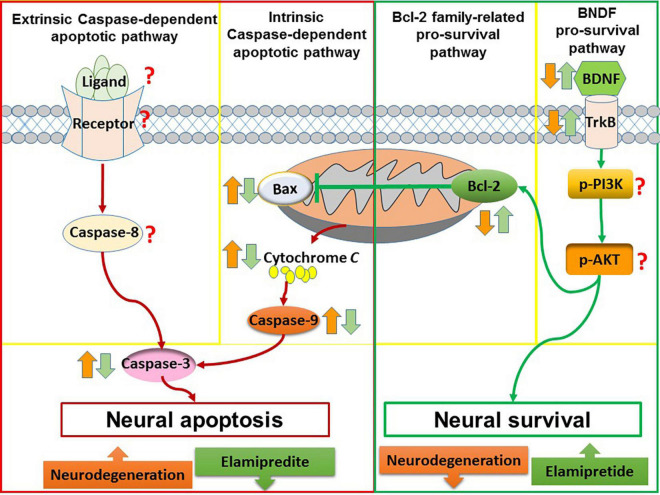
The anti-apoptotic and pro-survival effects of elamipretide. In neurodegeneration, both of intrinsic caspase-dependent apoptotic pathway and extrinsic caspase-dependent apoptotic pathway are overactive, as evidenced by the increased of apoptotic factors such as extrinsic death ligand, extrinsic receptor, Bax, caspase-9, and caspase-3. In addition, survival signaling such as Bcl-2, BDNF, TrkB, PI3K, and AKT are reduced. The reviewed studies showed that Elamipretide could reduce the levels of intrinsic caspase-dependent pro-apoptotic factors (i.e., Bax, caspase-9, and caspase-3) as well as enhanced pro-survival factors (i.e., Bcl-2, BDNF, and TrkB), suggesting that Elamipretide might reduce neural apoptosis and enhance neural survival against neurodegeneration. However, the effects of elamipretide on neural extrinsic caspase-dependent apoptotic pathway as well as on the levels of PI3K, AKT have maintained unclear.

Overall, elamipretide appears to reduce neural apoptosis and enhance neural survival in neurodegenerative disorders. A possible explanation is that elamipretide can increase the interaction between cytochrome *c* and cardiolipin, reducing in cytosolic cytochrome *c* release and inactivating of the mitochondria-mediated apoptotic pathway (Dai et al., 2017; Michael Seganish et al., 2017). Furthermore, due to its anti-inflammatory and antioxidant effects, elamipretide may reduce death stimuli, further inhibiting neural apoptosis. Integrating these findings with the previous neural apoptosis theories, a diagram (Figure 3) was drawn that suggests that elamipretide cannot only inhibit neural apoptotic pathways but also enhance neural survival pathways against neurodegeneration.

## Limitations and Perspective

Several limitations need to be considered in this review. The number of reviewed studies is limited. Thus, the reviewed findings need to be supported by further studies. Besides, we included English publications with full texts, which might have missed evidence reported by conference abstracts, non-English papers, and unpublished papers.

Although the *in vivo* and *in vitro* models used in the included studies typically mimic the neurodegenerative mechanisms, each model can just reflect certain features, and none of those models can completely represent all features of human neurodegenerative diseases (von Bohlen Und Halbach, 2005; Drummond and Wisniewski, 2017). In particular, Aβ-incubated cell and mutant Aβ-transfected cell models in the included studies can mimic neurons with toxic Aβ accumulation in Alzheimer’s disease. However, these *in vitro* models might ignore the pathological effects of the other components in the human brain, including glial roles (Drummond and Wisniewski, 2017). MPTP rodent model is one of the most useful Parkinsonian models that mimic neural mitochondrial dysfunction, but fail to mimic the production of Lewy bodies which are typically observed in Parkinson’s disease (von Bohlen Und Halbach, 2005). The limitations of preclinical models of neurodegeneration make the review findings hereby cannot be confirmed, requiring additional evidence.

**FIGURE 4:**
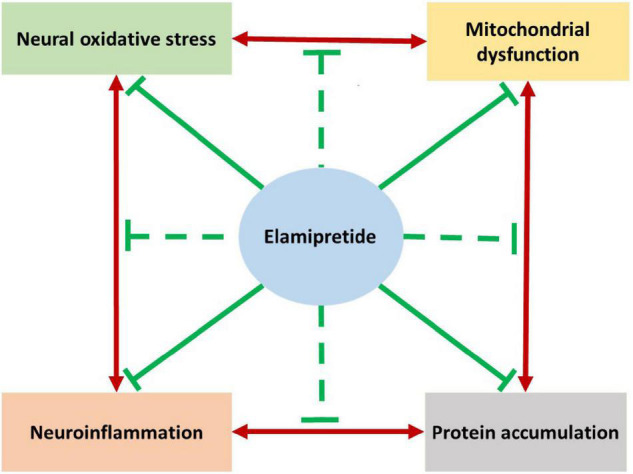
The hypothesized summarizes the neuroprotective effects of elamipretide on neurodegeneration. Previous studies reported that the underlying mechanisms of neurodegeneration are the complicated cause-consequences relationships among mitochondrial dysfunction, oxidative stress, neuroinflammation and protein accumulation. As reviewed, Elamipretide could prevent mitochondrial dysfunction promoted by protein accumulation, oxidative stress and neuroinflammation. Oxidative stress and secondary oxidative stress induced by mitochondrial dysfunction are observed to be suppressed by elamipretide. Furthermore, elamipretide appeared to reduce both primary neuroinflammation and protein accumulation-induced secondary neuroinflammation. Studies also reported that elamipretide could reduce the toxic protein accumulation in the brain with neurodegenerative disorders. Together, we might hypothesize that elamipretide not only prevent each mechanism of neurodegeneration but also interrupt their relationships. This hypothesis suggests the therapeutic effects of elamipretide to slow down the progression of neurodegenerative disorders.

The complicated pathogenesis of neurodegeneration is the greatest challenge of treatment. As reviewed, the elamipretide has neuroprotective effects in *in vivo* and *in vitro* studies. Based on the current evidence, we hypothesize that elamipretide can interrupt the interplay relationship among mitochondrial dysfunction, oxidative stress, neuroinflammation, and protein accumulation in neurodegeneration (Figure 4). However, due to insufficient data, several issues remained uncertain, expressing the need for further studies. First, the available evidence supports the benefits of elamipretide on secondary neural mitochondrial dysfunction promoted by protein accumulation, oxidative stress, and neuroinflammation but cannot indicate whether elamipretide can attenuate primary mitochondrial dysfunction, which is common in genetic neurodegeneration. Additionally, the effects of elamipretide on neural mitophagy, consisting of alterations in dysfunctional mitochondria detectors, autophagosomal activities, and lysosomal functions, are unclear. Second, although elamipretide is reported to directly counteract primary neuroinflammation, as observed in preclinical models, the effects of elamipretide on secondary neuroinflammation are not known. Finally, the current studies provided that Elamipretide could inactivate intrinsic mitochondrial apoptotic pathway factors (cytochrome c, Bax, caspase-9, caspase-3), but the effects of Elamipretide on the extrinsic neural apoptotic pathway are unclear.

It should be noted that there are several mitochondria-targeted compounds, which elamipretide and MitoQ are considered as therapeutics of neurodegeneration (Chaturvedi and Beal, 2008). In preclinical studies, MitoQ could reduce the toxic Aβ accumulation, mitochondrial dysfunction, and oxidative stress in models of Alzheimer’s disease (McManus et al., 2011). In the MPTP model of Parkinson’s disease, MitoQ has been shown to reduce neural apoptosis against MPTP-induced toxicity (Ghosh et al., 2010). However, MitoQ failed to show its benefits in Parkinsonian patients in clinical trials (Snow et al., 2010). As mentioned above, preclinical data also provide that elamipretide has the similar benefits on neurodegenerative mechanisms. However, elamipretide is absorbed by mitochondria independent with mitochondrial membrane potential, which is superior to the other mitochondrial-targeted drugs such as MitoQ (Dai et al., 2017), suggesting that elamipretide might be the promising compound to apply for the human neurodegeneration. In addition, a previous study reported that the combination of elamipretide and another mitochondria-targeted compound, Mdivi-1, exhibited superior therapeutic effects than a single treatment with elamipretide (Reddy et al., 2018). This result suggested that a combination treatment among mechanism-based compounds might be a research issue for further studies.

Elamipretide has been entered into clinical studies on cardiovascular diseases and myopathy. However, up to now, there is no clinical study that has analyzed the neuroprotective effects of Elamipretide in humans. Of note, previous studies showed that Elamipretide could attenuate the memory and learning deficits in animal studies with cognitive impairments (Wu et al., 2016, 2017). We hypothesized that elamipretide might improve both symptoms and mechanisms of neurodegenerative disorders. Further clinical studies are required to confirm the neuroprotective effects of elamipretide in patients with neurodegenerative disorders. Furthermore, the pharmacokinetics need to be clarified to find the optimal administration of elamipretide on neurodegenerative treatment.

## Conclusion

Our review aimed to systematically map the underlying mechanisms of elamipretide in neurodegenerative disorders. In both *in vivo* and *in vitro* models of neurodegenerative disorders, elamipretide treatment exhibited therapeutic effects against neurodegenerative mechanisms. The neuroprotective effects of elamipretide in neurodegeneration included attenuations in neural mitochondrial function, neural oxidative stress, neuroinflammation, protein accumulation, and neural apoptosis. Further preclinical studies should address the neuroprotective effects of elamipretide in both single treatment and combined treatment on the mentioned mechanisms of neurodegeneration. Additionally, clinical studies are required to evaluate the pharmacokinetics and pharmacodynamics of elamipretide in patients with neurodegenerative disorders.

## Author Contributions

NTN, Z-YC, and S-DL contributed to the conceptualization. NTN drafted the manuscript. YJL, S-YX, VBK, Z-YC, and S-DL edited and revised the manuscript. All authors approved the final version of the manuscript.

## Conflict of Interest

The authors declare that the research was conducted in the absence of any commercial or financial relationships that could be construed as a potential conflict of interest.

## Publisher’s Note

All claims expressed in this article are solely those of the authors and do not necessarily represent those of their affiliated organizations, or those of the publisher, the editors and the reviewers. Any product that may be evaluated in this article, or claim that may be made by its manufacturer, is not guaranteed or endorsed by the publisher.
